# Measurement properties of the interest in health scale among community-dwelling older adults in Japan: Verification of the 12-item, 6-item, and 4-item versions of the interest in health scale

**DOI:** 10.1016/j.pmedr.2026.103549

**Published:** 2026-06-20

**Authors:** Takuya Yamada, Yuta Nemoto, Noriko Takeda, Yoshinori Kitabatake, Kazushi Maruo, Hirono Ishikawa, Yoshiharu Fukuda, Takashi Arao

**Affiliations:** a Graduate School of Public Health, Teikyo University, 2-11-1 Kaga, Itabashi-ku, Tokyo 173-8605, Japan; b Tokyo Metropolitan Institute for Geriatrics and Gerontology, 35-2 Sakae-cho,Itabashi-ku, Tokyo, 173-0015, Japan; c School of Public Health, The University of Queensland, 266 Herston Rd, Herston QLD 4006, Australia; d Department of Preventive Medicine and Public Health, Tokyo Medical University, 6-1-1 Shinjuku, Shinjuku-ku, Tokyo 160-8402, Japan; e School of Health Innovation, Kanagawa University of Human Services, 3-25-10 Tonomachi, Kawasaki-ku, Kawasaki-shi, Kanagawa 210-0821, Japan; f Center for Promotion of Higher Education, Kogakuin University, 2665-1 Nakano, Hachiouji-shi, Tokyo 192-0015, Japan; g Department of Health Sciences, Saitama Prefectural University, 820 Sannomiya, Koshigaya-shi, Saitama 343-8540, Japan; h Faculty of Medicine, University of Tsukuba, 1-1-1 Tennoudai, Tsukuba-shi, Ibaraki 305-8575, Japan; i Physical Fitness Research Institute, Meiji Yasuda Life Foundation of Health and Welfare, 150 Tobuki, Hachiouji-shi, Tokyo 192-0001, Japan

**Keywords:** Interest in health, Scale validation, Older people, Health inequalities, Behavior science, Health promotion, Japan

## Abstract

**Objective:**

No validated scale currently evaluates “interest in health” among older adults. This cross-sectional study assessed the structural validity, internal consistency, and construct validity of the 12-item, 6-item, and 4-item Interest in Health Scale (IHS) in community-dwelling older adults.

**Methods:**

A mail survey was conducted in 2024 with 7203 independent older adults in Tsuru City, Japan. Structural validity was examined using exploratory and confirmatory factor analyses. Construct validity was assessed by analyzing the associations between IHS scores and health outcomes. Internal consistency was evaluated using Cronbach's α.

**Results:**

Confirmatory factor analysis supported the original three-factor structure of the 12-item IHS (comparative fit index = 0.965), which showed good internal consistency (α = 0.80). Shortened versions strongly correlated with the original version (*r* ≥ 0.85). Lower IHS scores were significantly associated with adverse health behaviors and outcomes, including physical inactivity and higher frailty risk (*p* < 0.01).

**Conclusions:**

The 12-item IHS is a valid and reliable measure for older adults, and its shortened versions may serve as practical screening tools by reducing response burden while maintaining psychometric integrity. The choice depends on balancing brevity for rapid screening with the need for a comprehensive understanding of health values to tailor individual interventions.

## Highlights

• The 12-item version of Interest in Health Scale showed high internal consistency and construct validity.
• The 12-item version replicated the original three-factor structure.
• Both the 4-item and 6-item shortened versions of the Interest in Health Scale showed acceptable utility.
• The 6-item version maintained the original three-factor structure.
• The 4-item version maximized internal consistency.

## 1 Introduction

Health disparities disproportionately affect socially disadvantaged groups and impose substantial societal burden, including reduced labor productivity and increased healthcare costs (Commission on Social Determinants of Health, 2008). Thus, reducing inequities in health outcomes and preventive behaviors is a public health priority.

Socioeconomic conditions (Bammert et al., 2024; Manfredi et al., 2025; Patel et al., 2007) and differences in health literacy (Sanders et al., 2009; Tamayo-Fonseca et al., 2023) contribute to health disparities; nevertheless, these factors do not fully explain heterogeneity in preventive behaviors (Gupta et al., 2025; Manfredi et al., 2025). Despite the availability of evidence-based services and health-related information, the underuse of preventive services and difficulty in sustaining health behaviors remain widely recognized challenges. These phenomena have been discussed within behavioral and psychological frameworks, including the intention–behavior gap (Faries, 2016; Feil et al., 2023).

Psychological factors are central to health behavior research and theoretical models of preventive actions and have been proposed as explanations for the intention–behavior gap (Armitage and Conner, 2001). In particular, psychological constructs such as health consciousness (Espinosa, 2023; Gould, 1990; Iversen and Kraft, 2006; Xue et al., 2020) and health values (Lau et al., 1986; Rosengard et al., 2001; Shi et al., 2004) have been identified as correlates of health behaviors. Health consciousness reflects awareness of health (Gould, 1990), and health values reflect the priority placed on health relative to other life domains (Lau et al., 1986; Rosengard et al., 2001). Health literacy refers to the ability to process health-related information (Sanders et al., 2009; Tamayo-Fonseca et al., 2023). However, these constructs focus on specific cognitive abilities or motivational states and do not directly address the broader psychological orientation reflecting the degree to which individuals maintain interest in health throughout their daily lives. Interest in health has been proposed as such an orientation (Fukuda et al., 2024), representing an attempt to operationalize a multidimensional psychological disposition integrating health consciousness, motivation to maintain health, and values placed on prioritizing health over other life domains. Interest in health thus reflects the personal relevance of health in everyday life. Interest in health may function as an upstream psychological orientation that shapes the engagement of individuals in seeking health-related information, utilizing preventive services, and exhibiting health behaviors. In super-aging societies such as Japan, identifying individuals with low interest in health may help reduce health disparities.

Instruments directly evaluating the multidimensional nature of interest in health remain limited. Existing scales such as the Health Consciousness Scale (Gould, 1990) and Health Orientation Scale (Snell et al., 1991) primarily capture attentiveness to health or broad personality traits rather than integrating consciousness, motivation, and value prioritization. Despite theoretical associations with reduced engagement in preventive actions (Fukuda et al., 2024), empirical evidence linking interest in health to actual behaviors and outcomes in older populations remains scarce (Iwase and Hosokawa, 2025; Wakabayashi et al., 2023). This constitutes a critical issue for older adults, who carry higher risks of multimorbidity, frailty, and functional decline (Beard et al., 2016). Accordingly, the Interest in Health Scale (IHS) for general adults was developed to address these gaps (Ozawa et al., 2021); however, the psychometric properties of the 12-item IHS, including its shortened 6-item and 4-item versions, have been established only in general adults (Ozawa et al., 2021; Yamada et al., 2025).

To address these methodological and empirical gaps, the present study aimed (i) to evaluate the cross-validity of the 12-item IHS in community-dwelling older adults, (ii) to investigate its associations with health behaviors and outcomes, and (iii) to assess the cross-validity and comparative utility of the shortened 6-item and 4-item versions of the IHS and ascertain the most feasible tool for older adults.

## 2 Methods

### 2.1 Study design and participants

This cross-sectional study adhered to the COnsensus-based Standards for the selection of health Measurement INstruments (COSMIN) guidelines (Mokkink et al., 2010) (Supplementary Table S1). In January 2024, a city-wide census was conducted with 7203 older adults aged ≥65 years in Tsuru City, Yamanashi Prefecture, who were living independently and did not require long-term care services. Tsuru is a rural city located approximately 90 km west of Tokyo and surrounded by mountains, with a population of approximately 28,000 as of April 2025 and an aging rate of 31.2% as of January 2024.

**Fig. 1:**
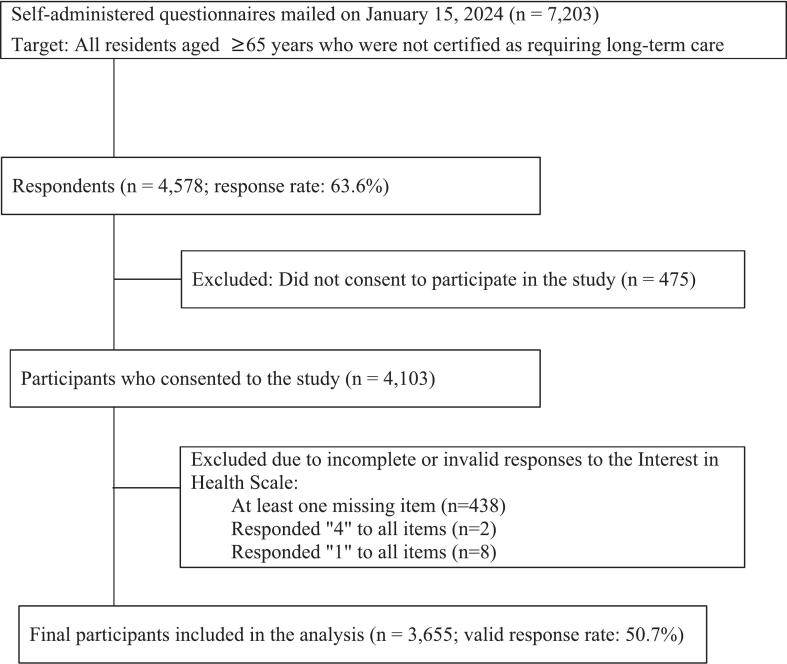
Flow diagram of the participant selection process in the 2024 survey of community-dwelling older adults in Tsuru City, Japan.

A self-administered questionnaire was mailed to all eligible older adults. Data derived from 3655 community-dwelling older adults who provided informed consent and had no missing IHS data (valid response rate: 50.7%) were analyzed (Fig. 1). This sample size sufficiently exceeded general recommendations for psychometric validation and hypothesis testing.

This study was conducted in accordance with the principles embodied in the Declaration of Helsinki and was approved by the Ethics Review Committee of Tokyo Metropolitan Institute of Gerontology (approval no. R23–057; approval date: September 28, 2023). Written explanations regarding the study purpose, voluntary participation, and data protection were provided to the participants; checking the “I agree” box constituted informed consent.

### 2.2 Measurements

#### 2.2.1 IHS

The 12-item IHS (Ozawa C, Ishikawa H, Kato M, Fukuda Y, 2021) consists of three subscales (Supplementary Table S2), with items being rated on a 4-point Likert scale ranging from “agree” and “somewhat agree” to “somewhat disagree” and “disagree.” Health value subscale items are reverse-scored (0 = “agree” to 3 = “disagree”). The total score ranges from 0 to 36, with higher scores indicating greater interest in health. In the present study, the 6-item and 4-item versions of the IHS adopted directly from a previous study (Yamada et al., 2025) were evaluated to determine their applicability to older adults.

#### 2.2.2 Sociodemographic characteristics and health-related variables

Data on sociodemographic characteristics, including age, sex, educational attainment, marital status, living arrangement, employment status, and perceived financial status, were obtained. These variables were examined to describe the participants' background.

Health-related variables theoretically associated with interest in health were assessed to examine construct validity and explore the associations between IHS scores and health outcomes. Health behaviors encompassed smoking status, alcohol consumption, dietary habits, physical activity, and frequency of seeking health-related information. Smoking status was categorized into never smokers, former smokers, and current smokers (Murakami et al., 2007). The frequency of alcohol consumption was categorized into “nothing,” “1–2 days per week,” and “≥3 days per week.” Dietary habits were assessed according to the frequency of eating balanced meals (staple food, main dish, and side dishes) at least twice a day (The Ministry of Health, Labour and Welfare of Japan, n.d). Physical activity was evaluated using the International Physical Activity Questionnaire–Short Form (Tomioka et al., 2011), and the participants were categorized according to total physical activity (min/week) into those meeting the physical activity guidelines (≥150 min/week) and those who did not (Bull et al., 2020). The frequency of seeking health-related information was dichotomized into “once a month or more” and “less than once a month” (Karasawa et al., 2025).

Health status was assessed using self-rated health and frailty risk. Self-rated health was evaluated by asking the single question, “How would you describe your usual health condition?”, with four response options being presented. Subsequently, responses were dichotomized into “good health” (very healthy or somewhat healthy) and “poor health” (not very healthy or not healthy) (Ichida et al., 2009). Frailty risk was evaluated using the 25-item Kihon Checklist (Satake et al., 2016); a total score of 0–3, 4–7 and ≥ 8 points indicated robust, pre-frailty, and frailty participants, respectively.

### 2.3 Statistical analysis

An exploratory factor analysis (EFA) was conducted using maximum likelihood estimation with Promax rotation to clarify the factor structure of the 12-item IHS. The number of factors was determined based on the Kaiser–Guttman criterion (eigenvalues >1.0) and the scree plot.

A confirmatory factor analysis (CFA) was also performed using the comparative fit index (CFI), Tucker–Lewis index (TLI), root mean square error of approximation (RMSEA), and standardized root mean squared residual (SRMR) to examine model fit. Internal consistency reliability was assessed using Cronbach's α.

The relationships between the 12-item, 6-item, and 4-item IHS scores and various health-related variables were explored to analyze their associations with health outcomes and compare the utility of the shortened versions of the IHS. The mean IHS scores of groups categorized by health behaviors and status were calculated, and differences in mean scores among groups were examined using one-way analysis of variance. These analyses were also conducted to evaluate the construct validity of the scales. Additionally, complete response rates among all consenting respondents were calculated for each version to assess the feasibility of the shortened versions.

All statistical analyses were performed using R version 4.4.1 (R Foundation for Statistical Computing, Vienna, Austria), with “psych” and “lavaan” packages being utilized for psychometric evaluations. Statistical significance was set at a two-sided *p* value of <0.05.

## 3 Results

The characteristics of 3655 community-dwelling older adults included in the analysis are summarized in Table 1. The mean age was 74.8 years (standard deviation: 6.7), with female and married participants accounting for 52.4% and 72.5%, respectively. Overall, 86.0% of participants lived with others.

**Table 1:** Sociodemographic characteristics of study participants in Tsuru City, Japan in January 2024 (*n* = 3655).

	n (%) or mean (SD)
Age, years [mean (SD)]	74.8 (6.7)
Sex	
Male	1719 (47.6%)
Female	1896 (52.4%)
Educational attainment	
≤ 9 years	677 (18.7%)
10–12 years	1896 (52.2%)
≥ 13 years	1059 (29.2%)
Marital status	
Married	2631 (72.5%)
Divorced	178 (4.9%)
Widowed	682 (18.8%)
Never married	139 (3.8%)
Living arrangement	
Living with other	3112 (86.0%)
Living alone	508 (14.0%)
Employment status	
Employment	1336 (36.9%)
Unemployment	2283 (63.1%)
Subjective economic status	
Poor	829 (22.9%)
Neither one	1707 (47.1%)
Good	1088 (30.0%)

SD, standard deviation

The EFA conducted on the 12 IHS items confirmed a three-factor structure. Maximum likelihood estimation with Promax rotation produced standardized factor loadings that clearly clustered into three distinct but correlated factors—namely, health consciousness, health motivation, and health value (Table 2). Factor loadings ranged from 0.45 to 0.81.

**Table 2:** Exploratory factor analysis of the 12-item Interest in Health Scale among community-dwelling older adults in Tsuru City, Japan in January 2024 (n = 3655).

Items	Consciousness	Motivation	Value	*h*^*2*^	6-item†	4-item†
1. I'm very self-conscious about my health	0.81	−0.07	−0.00	0.59	✓	✓
2. I'm interested in information about my health	0.68	0.07	0.03	0.55	✓	✓
3. I pay attention to changes in my health condition	0.65	0.12	0.03	0.55		
4. I am more health conscious than people around me	0.63	0.20	−0.11	0.59		
5. We should spend some extra time for health	0.18	0.56	0.04	0.50	✓	✓
6. I am willing to spend some extra money for my health	−0.04	0.72	0.01	0.47		
7. I want to put health first in my living	0.06	0.65	0.06	0.49	✓	✓
8. I do everything I can to stay healthy	0.03	0.67	−0.07	0.47		
9. Hobbies and leisure activities are more important than health*	−0.12	0.04	0.58	0.32		
10. Work and income are more important than health*	−0.14	0.08	0.67	0.43	✓	
11. Rather than prevent illness, it is just to cure when I get sick*	0.10	0.01	0.58	0.38	✓	
12. I worry about my health only when I get sick*	0.16	−0.11	0.45	0.24		

Exploratory factor analysis used maximum likelihood estimation and Promax rotation.

*h*^*2*^ represents the commonality (proportion of variance accounted for by the three factors).

Items were rated on a 4-point scale (3 = *Agree*, 2 = *Somewhat agree*, 1 = *Somewhat disagree*, 0 = *Disagree*).

Items marked with an asterisk (*) are reversed score (0 = *Agree*, 1 = *Somewhat agree*, 2 = *Somewhat disagree*, 3 = *Disagree*).

† Checks (✓) indicate items included in the shortened versions. To ensure consistency with the original scale developed for general adults, we continued to select these items for the shortened versions, although they did not show the highest factor loadings in the present study.

The CFA supported the three-factor model (Supplementary Fig. S1), revealing acceptable model fit indices (CFI = 0.965, TLI = 0.954, SRMR = 0.058, RMSEA = 0.083). In the CFA, all factor loadings were statistically significant and high (range: 0.58–0.85). Health consciousness strongly correlated with health motivation (*r* = 0.80).

Two shortened versions of the IHS were evaluated. Items for these two shortened versions were adopted directly from a previous study (Yamada et al., 2025) to ensure comparability with general adult populations. Consequently, items 5 and 7 for the “health motivation” factor did not show the highest factor loadings (Table 2).

The psychometric properties of IHS versions are presented in Supplementary Table S3. Cronbach's α was 0.80 for the 12-item version, 0.68 for the 6-item version, and 0.77 for the 4-item version. Both shortened versions showed strong positive correlations with the original 12-item version (*r* = 0.94 and 0.85 for the 6-item and 4-item versions, respectively). The complete response rates among all consenting respondents (*n* = 4103) were higher with the 4-item (94.9%) and 6-item (92.7%) versions than with the 12-item version (89.3%) (Supplementary Table S3).

The associations between the 4-item, 6-item, and 12-item IHS scores and health-related variables are presented in Table 3. Participants with healthier lifestyles and better health status attained significantly higher IHS scores across almost all categories (*p* < 0.01). For instance, participants who met the physical activity guidelines (≥150 min/week) exhibited significantly higher scores than those who did not; this trend was observed with both the 12-item version (26.7 vs. 24.9) and 4-item version (9.6 vs. 8.4). Similarly, nonsmokers and participants consuming balanced meals almost daily showed significantly higher interest in health. A clear gradient in relation to health outcomes was also observed: IHS scores progressively decreased as frailty status worsened. For the 12-item version, the mean score was 27.3 in robust participants, which decreased to 25.6 and 24.4 in pre-frailty and frailty participants, respectively. For the two shortened versions, such stepwise decline was replicated; however, a notable difference in alcohol consumption was observed: the 12-item (*p* = 0.01) and 6-item (*p* = 0.02) versions exhibited significant associations, whereas the 4-item version did not show a statistically significant difference (*p* = 0.35).

**Table 3:** Comparison of the Interest in Health Scale (IHS) scores by health-related variables among community-dwelling older adults in Tsuru City, Japan in January 2024 (n = 3655).

Variables	n (%)	IHS 12-item	*p value*	IHS 6-item	*p value*	IHS 4-item	*p value*
Total score	3655 (100)	25.8 (5.6)	–	13.7 (3.0)	–	9.0 (2.3)	–
Current alcohol consumption frequency							
Nothing	2153 (60.6)	26.1 (5.4)	0.01	13.8 (2.9)	0.02	9.1 (2.3)	0.35
< 3 days per week	646 (18.2)	25.9 (5.8)		13.6 (3.1)		8.9 (2.4)	
≥ 3 days per week	756 (21.3)	25.4 (5.6)		13.4 (3.0)		9.0 (2.2)	
Current smoking							
Never smoked	1918 (53.8)	26.6 (5.3)	<0.01	14.1 (2.8)	<0.01	9.2 (2.2)	<0.01
Former smoker	1257 (35.3)	25.6 (5.6)		13.5 (3.0)		9.0 (2.2)	
Smoker	387 (10.9)	23.2 (6.0)		12.2 (3.2)		8.1 (2.6)	
Frequency of balanced meals per day (≥2 times/day)							
Almost every day	3149 (86.4)	26.1 (5.5)	<0.01	13.8 (2.9)	<0.01	9.1 (2.2)	<0.01
4–5 days a week	289 (7.9)	24.7 (5.6)		13.0 (3.0)		8.6 (2.3)	
2–3 days a week	143 (3.9)	24.5 (5.6)		13.0 (3.0)		8.5 (2.4)	
≤ 1 day a week	64 (1.8)	22.5 (6.6)		11.9 (3.5)		7.5 (3.0)	
Moderate-to-vigorous physical activity							
≥ 150 min/week	1894 (51.8)	26.7 (5.3)	<0.01	14.1 (2.8)	<0.01	9.3 (2.1)	<0.01
< 150 min/week	1761 (48.2)	24.9 (5.8)		13.2 (3.1)		8.6 (2.4)	
Frequency of Health Information Collection							
≥ once per month	1697 (50.4)	27.4 (4.9)	<0.01	14.5 (2.6)	<0.01	9.6 (1.9)	<0.01
< once per month	1668 (49.6)	24.4 (5.8)		12.9 (3.1)		8.4 (2.4)	
Self-rated health							
Good	2727 (75.3)	26.3 (5.4)	<0.01	13.9 (2.9)	<0.01	9.2 (2.2)	<0.01
Poor	895 (24.7)	24.8 (5.7)		13.1 (3.1)		8.6 (2.4)	
Frailty status							
Robust	1197 (39.4)	27.3 (5.2)	<0.01	14.4 (2.7)	<0.01	9.5 (2.1)	<0.01
Pre-frailty	1121 (36.9)	25.6 (5.5)		13.6 (2.9)		8.9 (2.3)	
Frailty	717 (23.6)	24.4 (5.8)		12.8 (3.1)		8.5 (2.4)	

Scores are presented as mean (standard deviation). *p value*s were calculated using one-way analysis of variance.

## 4 Discussion

This study sought to verify the cross-validity of the IHS in community-dwelling older adults and to compare the utility of its shortened versions. Two key findings emerged: (i) the 12-item IHS not only demonstrated high internal consistency and construct validity but also replicated the original three-factor structure (namely, health consciousness, health motivation, and health value) and (ii) both the 4-item and 6-item shortened versions of the IHS showed acceptable utility. The 6-item version maintained the original three-factor structure, whereas the 4-item version maximized internal consistency by focusing on health consciousness and motivation. These findings support the validity of both shortened versions for assessing interest in health among older adults. The appropriate version can be selected based on research or practice aim.

The first major finding supports the applicability of the IHS to older adults. The EFA and CFA supported the original three-factor structure identified in the previous study involving adults (Ozawa C, Ishikawa H, Kato M, Fukuda Y, 2021), suggesting that the concept of “interest in health” is multifaceted among older adults and encompasses not only health consciousness and motivation but also health prioritization within one's value system. Its internal consistency (Cronbach's α = 0.80) and associations with health-related variables support its validity.

Nevertheless, factor loadings for health motivation differed from those found in a previous study on general adults (Yamada et al., 2025). Among younger adults, items related to time allocation (e.g., “We should spend some extra time for health”) or health prioritization (e.g., “I want to put health first in my living”) had the highest loadings. In contrast, among older adults included in the present study, items reflecting specific commitments (e.g., “I am willing to spend some extra money for my health,” “I do everything I can to stay healthy”) showed higher factor loadings than time-related items. This discrepancy likely reflects differences in life stage. Older adults, many of whom have retired, may have more discretionary time, making spending time a less distinguishing feature of high motivation. Conversely, given that financial resources are limited and the risk of diseases increases with age (Beard et al., 2016), the willingness to invest money or exert concrete efforts is a stronger indicator of motivation. Although the original items (time and priority) were retained for the two shortened versions in the present study to ensure comparability with the adult version, these findings suggest that the manifestation of health motivation evolves with age.

With respect to internal consistency, the 4-item version (α = 0.77) outperformed the 6-item version (α = 0.68). This difference stems from the low consistency of the “health value” factor among older adults (α = 0.51). The poor performance of the “health value” factor may be attributable to conceptual and methodological factors. Conceptually, prioritizing health over work or leisure may become less distinguishing after retirement because health becomes a universal goal. Methodologically, reverse-scored items for the “health value” factor may have introduced cognitive burden and caused response inconsistencies or confusion (Swain et al., 2008), particularly among older adults (Carlson et al., 2011). Notably, the shortened versions significantly improved the complete response rates (94.9% and 89.3% for the 4-item and 12-item versions, respectively), showing superior feasibility. Thus, the 4-item version is recommended as a large-scale screening tool for older adults, whereas the 6-item version is a valid option for clinical interventions requiring a multifaceted assessment of the original three-factor construct.

The construct validity analysis revealed that lower IHS scores were associated with physical inactivity, smoking, and higher frailty risk, highlighting the potential of the IHS to identify individuals indifferent to health, a group that is often overlooked by conventional health education. Nonetheless, the results regarding alcohol consumption require careful interpretation. Unlike previous studies on general adults (Ozawa et al., 2021; Yamada et al., 2025), the present study identified significant associations with the 12-item and 6-item versions. This discrepancy may stem from methodological and contextual differences. Methodologically, our substantially larger sample size provided greater statistical power for the detection of smaller effect sizes. Contextually, given the increased health risks associated with aging, interest in health among older adults may be more directly reflected in concrete actions, such as self-restraint in alcohol consumption. The 4-item version showed no such association, suggesting that limiting alcohol consumption may require the “health value” aspect. Despite this, the 4-item IHS remains useful because it is significantly associated with frailty status and physical inactivity. Integrating it into routine health checkups may help healthcare providers in identifying older adults disengaged from health behaviors, thus facilitating tailored interventions, such as outreach-based programs aimed at reducing late-life health disparities.

This study's strengths include its large sample size of 3655 community-dwelling older adults, providing high statistical power to detect associations with health behaviors. Furthermore, we demonstrated that the shortened 4-item IHS offers superior feasibility with a 94.9% complete response rate, confirming its practical utility for large-scale geriatric screenings.

This study has some limitations. First, the cross-sectional design precludes causal inferences, specifically regarding whether higher interest in health leads to better health outcomes or vice versa. Longitudinal studies should be conducted to investigate whether health outcomes would improve over time upon implementation of interventions that increase interest in health. Second, this study was conducted in a single municipality in Japan, limiting the generalizability of the findings to other settings, including urban environments or populations with different cultural backgrounds and healthcare systems. Interest in health may vary across social and health care contexts. Future cross-cultural studies should examine the psychometric properties of the IHS across diverse populations. Third, the use of self-administered questionnaires raises the possibility of social desirability bias, which may have attenuated differences in responses. Fourth, nonrespondents possibly had lower interest in health than respondents, introducing potential sampling bias. This restricted variance may attenuate psychometric estimates and associations with health behaviors. Future studies should estimate nonrespondent characteristics and adjust for potential biases to further validate the IHS.

## 5 Conclusion

The 12-item IHS demonstrated high reliability and validity in community-dwelling older adults, supporting its use as a measure of interest in health in this population. Both the 6-item and 4-item shortened versions of the IHS showed acceptable psychometric properties. Thus, the selection of the most appropriate version of the IHS (i.e., whether the 12-item, 6-item, or 4-item version) should depend on the project's specific purpose. The IHS can help identify older adults with lower interest in health who are at risk of disengaging from preventive services and healthier behaviors, thereby providing a basis for the development of targeted health promotion strategies for older adults.

## Declaration of generative AI and AI-assisted technologies in the manuscript preparation process

During the preparation of this work, the authors used Gemini (Google) and Editage (www.editage.jp) for English language editing and readability improvements. After using this tool/service, the authors reviewed and edited the content as needed and take full responsibility for the content of the published article.

## CRediT authorship contribution statement

**Takuya Yamada:** Writing – review & editing, Writing – original draft, Visualization, Software, Methodology, Investigation, Funding acquisition, Formal analysis, Data curation, Conceptualization. **Yuta Nemoto:** Writing – review & editing, Funding acquisition, Data curation, Conceptualization. **Noriko Takeda:** Writing – review & editing, Funding acquisition, Data curation. **Yoshinori Kitabatake:** Writing – review & editing, Project administration, Data curation. **Kazushi Maruo:** Writing – review & editing, Formal analysis. **Hirono Ishikawa:** Writing – review & editing, Methodology, Conceptualization. **Yoshiharu Fukuda:** Writing – review & editing, Methodology, Conceptualization. **Takashi Arao:** Writing – review & editing, Funding acquisition, Data curation.

## Funding

This work was supported by 10.13039/501100001691JSPS KAKENHI (JP21K11418, JP22H03436, JP23K19812) and 10.13039/100014475Pfizer Health Research Foundation. The views and opinions expressed in this article are those of the authors and do not necessarily reflect the official policy or position of the respective funding organizations. The funders had no role in the study design, data analysis and interpretation, writing of the article, or decision to submit the article for publication.

## Declaration of competing interest

The authors declare that they have no known competing financial interests or personal relationships that could have appeared to influence the work reported in this paper.

## Data Availability

Data will be made available on request.
