# Supplementary material for: Measurement properties of the interest in health scale among community-dwelling older adults in Japan: Verification of the 12-item, 6-item, and 4-item versions of the interest in health scale
Source: Prev Med Rep. 2026 Jun 20;68:103549. doi: 10.1016/j.pmedr.2026.103549 (PMC13316078; doi:10.1016/j.pmedr.2026.103549)
Supplement: Supplementary file 1 — Supplementary material [file mmc1.docx]

| **Supplementary Table A.1. Checklist based on the COSMIN guideline for reporting measurement properties of the Interest in Health Scale among community-dwelling older adults in Tsuru City, Japan in January 2024** | | | | |
| --- | --- | --- | --- | --- |
| Section / Item | Item name | Item description | Reported on Page # |  |
| I. General Reporting Recommendations | | | | |
| Title | | | | |
| T1 | Title | Identify the report as a study of one or more measurement properties of a specific PROM to measure a specified construct in a specified population. | 1 |  |
| Abstract | | | | |
| A1 | Objectives | Provide the specific objective(s) of the research, specifying (1) the name (and version, if relevant), and construct(s) of the PROM, (2) the measurement properties being evaluated, and (3) relevant study characteristics. | 3 |  |
| A2 | Design | Specify (details of the) study design used to evaluate the measurement properties. | 3 |  |
| A3 | Methods | Specify the methods for evaluating each measurement property. | 3 |  |
| A4 | Results | Provide the main results for all measurement properties evaluated. | 3 |  |
| A5 | Discussion/Conclusions | Provide a brief statement of the implications of the findings in the context of existing evidence on the PROM. | 3 |  |
| Introduction | | | | |
| I1 | PROM | Specify the name and, if relevant, the version, and construct(s) of the PROM. | 7 |  |
| I2 | Target population & context of use | Specify the target population and context of use that the PROM was designed for. | 7 |  |
| I3 | State of knowledge & Rationale | Provide a description of the current scientific knowledge (what is known and not known) regarding the measurement properties of the PROM. Explain why the new study is necessary. Provide citations for the original development paper(s). | 4-7 |  |
| I4 | Objectives | Provide the specific objective(s) of the research, specifying (1) the name (and version, if relevant) of the PROM, (2) the measurement properties being evaluated, and (3) relevant study sample characteristics. | 7-8 |  |
| General Methods | | | | |
| GM1 | Study design | Specify (details of the) study design used to evaluate the measurement properties. | 8 |  |
| GM2 | Participants | Specify how the study participants were selected. Specify the inclusion and exclusion criteria | 8 |  |
| GM3 | PROM details | Provide details about the original version of the PROM as well as of the PROM version being studied, specify the conceptual framework (reflective/formative model), details on the structure (the number of items and subscales), the language, response scale, recall period, direction of scoring, and scoring algorithm of the PROM. Specify how the PROM was administered (e.g., in what setting, mode of administration (e.g. paper, electronic) what instructions were given), including the country in which it is administered | 10 |  |

| **Supplementary Table A.1. Continued** | | | | |  |
| --- | --- | --- | --- | --- | --- |
| Section / Item | Item name | Item description | Reported on Page # | |  |
| GM4 | Additional data collection | Describe why and how other data was collected (e.g., construct and measurement properties of the comparator instruments, characteristics of groups being compared, and rationale for choosing groups), including mode of administration (e.g., paper, electronic). | 11-13 |  |  |
| GM5 | Time points procedures | Provide all time points of all measurements. | 8 |  |  |
| GM6 | Justification for sample size | Provide a rationale for the sample size for all measurement properties analyses (including subgroups). | 8-9 |  |  |
| GM7 | Statistical analyses | Describe the statistical analyses corresponding to all objectives (see measurement properties specific boxes). Describe the criteria for good measurement properties. Name the statistical package used and the version. | 13-15 |  |  |
| GM8 | Missing data | Describe approaches for dealing with missing data. | 9 |  |  |
| GM9 | Unplanned analysis | Specify analyses that were unplanned and their rationale. | N/A |  |  |
| General Results | | | | | |
| GR1 | Participant characteristics | Provide study participants' characteristics, specified per subgroup if applicable. | 15 | |  |
| GR2 | Sample size | Provide the total number of participants included in the study and the sample size for each analysis. | 15 | |  |
| GR3 | Missing data | Provide amount of (proportion or count) and reasons for missing data for each analysis for the PROM, and for any analyses of other outcome measurement instruments. | N/A | |  |
| GR4 | Results | Describe the results corresponding to all objectives (see measurement properties specific boxes). | 15-18 | |  |
| Discussion/conclusions | | | | | |
| DC1 | Measurement property evidence | Provide the main findings and if each measurement property is sufficient or insufficient and why. | 19 | |  |
| DC2 | Practical relevance | Discuss the practical relevance of the findings in terms of recommendations for (not) using the PROM. | 19 | |  |
| DC3 | Strengths and limitations | Discuss strengths and limitations of each study. For example, discuss if there were any potential biases in the study that could have impacted the results. | 24-26 | |  |
| DC4 | Generalizability | Discuss generalizability of the results. For example, discuss whether the results could be generalized to other populations given the sample studied. | 25 | |  |
| DC5 | Instrument changes | Discuss what modifications are needed to the existing PROM. | N/A | |  |
| DC6 | Future research | Describe new research questions or hypotheses generated from these findings, and provide/describe the research needed to answer those questions. | 25-26 | |  |
| DC7 | Conclusions | Provide the overall conclusions for the use of the PROM. | 26-27 | |  |

| **Supplementary Table A.1. Continued** | | | |  |
| --- | --- | --- | --- | --- |
| Section / Item | Item name | Item description | Reported on Page # |  |
| Other information | | | | |
| O1 | Conflict of interest | State any conflict of interest you may have related to the PROM. This may include any involvement in the development of the PROM or any commercial funding or profit. | 29 |  |
| II. Specific Reporting Recommendations | | | | |
| Structural Validity | | | | |
| SV1 | Rationale for approach | Provide a rationale for the approach (e.g., factor analysis, Item Response Theory (IRT)/Rasch analysis) used. | 13 |  |
| SV2 | Statistical analyses | Exploratory (EFA) or confirmatory factor analyses (CFA)  Describe the tested model (e.g., number of factors, which items included in which factor), method of estimation, type of correlation matrix, and methods and criteria for good model fit.  The details of IRT/Rasch are omitted because they are not applicable. | 13-14 |  |
| SV3 | Results | EFA or CFA  For EFA: provide all factor loadings, eigenvalues and % variance explained of the model reflecting the original PROM structure and best-fitting model if applicable.  For CFA: provide all factor loadings and results for model fit indices of the model reflecting the original PROM structure and best-fitting model, if applicable. | 15-16 |  |
| Internal Consistency | | | | |
| IC1 | Statistical analyses | Provide evidence for the unidimensionality of the PROM (subscales) and provide evidence of lack of local item dependence. Describe statistical methods used to calculate internal consistency. | 14 |  |
| IC2 | Results | Provide internal consistency results for each unidimensional scale or subscale separately. | 17 |  |
| Hypotheses Testing for Construct Validity | | | | |
| ConV1 | Hypotheses | State hypotheses, and provide the rationale for each hypothesis. | 7-8 |  |
| ConV2 | Statistical analyses | Specify all statistical methods used to test the hypotheses. | 14 |  |
| ConV3 | Results | Provide all results and specify if each result is in accordance with its hypothesis. | 17-18 |  |

| **Supplementary Table A.2. The 12-item Interest in Health Scale and its shortened versions used in this study** | | | | | | |
| --- | --- | --- | --- | --- | --- | --- |
| Health Consciousness | 3 | 2 | 1 | 0 | 6-item* | 4-item* |
| 1. I’m very self-conscious about my health | Agree | Somewhat agree | Somewhat disagree | Disagree | ✓ | ✓ |
| 2. I’m interested in information about my health | Agree | Somewhat agree | Somewhat disagree | Disagree | ✓ | ✓ |
| 3. I pay attention to changes in my health condition | Agree | Somewhat agree | Somewhat disagree | Disagree |  |  |
| 4. I am more health conscious than people around me | Agree | Somewhat agree | Somewhat disagree | Disagree |  |  |
| Health Motivation | 3 | 2 | 1 | 0 |  |  |
| 5. We should spend some extra time for health | Agree | Somewhat agree | Somewhat disagree | Disagree | ✓ | ✓ |
| 6. I am willing to spend some extra money for my health | Agree | Somewhat agree | Somewhat disagree | Disagree |  |  |
| 7. I want to put health first in my living | Agree | Somewhat agree | Somewhat disagree | Disagree | ✓ | ✓ |
| 8. I do everything I can to stay healthy | Agree | Somewhat agree | Somewhat disagree | Disagree |  |  |
| Health Value | 0 | 1 | 2 | 3 |  |  |
| 9. Hobbies and leisure activities are more important than health | Agree | Somewhat agree | Somewhat disagree | Disagree |  |  |
| 10. Work and income are more important than health | Agree | Somewhat agree | Somewhat disagree | Disagree | ✓ |  |
| 11. Rather than prevent illness, it is just to cure when I get sick | Agree | Somewhat agree | Somewhat disagree | Disagree | ✓ |  |
| 12. I worry about my health only when I get sick | Agree | Somewhat agree | Somewhat disagree | Disagree |  |  |

*This column's check indicates items in the 6-item or 4-item short versions.

Scaled Comparative Fit Index (CFI) ＝0.965，Scaled Tucker-Lewis Index (TLI) = 0.954,

Scaled Root Mean Square Error of Approximation (RMSEA) ＝0.083, Standardized Root Mean Square Residual (SRMR) = 0.058

0.58

0.85

0.67

0.58

0.72

0.80

0.73

0.82

0.83

0.84

0.80

0.31

0.26

0.80

0.82

Health Consciousness

Health Motivation

Health Value

I’m very self-conscious about my health

I’m interested in information about my health

I pay attention to changes in my health condition

I am more health conscious than people around me

We should spend some extra time for health

I am willing to spend some extra money for my health

I want to put health first in my living

I do everything I can to stay healthy

Hobbies and leisure activities are more important than health

Work and income are more important than health

Rather than prevent illness, it is just to cure when I get sick

I worry about my health only when I get sick

**Supplementary Fig A. 1. Results of confirmatory factor analyses for the 12-item Interest in Health Scale among community-dwelling older adults in Tsuru City, Japan (n = 3,655) a**

**Supplementary Table A.3. Comparison of psychometric properties and feasibility among the three versions of the Interest in Health Scale (IHS)**

| Variables | IHS 12-item | IHS 6-item | IHS 4-item |
| --- | --- | --- | --- |
| Complete response rate % (n/Total respondents with consent) | 89.3 (3,665/4,103) | 92.7 (3,804/4,103) | 94.9 (3,894/4,103) |
| Cronbach's α for total scale | 0.80 | 0.68 | 0.77 |
| Cronbach's α for Health Consciousness subscale | 0.83 | 0.73 | 0.73 |
| Cronbach's α for Health Motivation subscale | 0.78 | 0.65 | 0.65 |
| Cronbach's α for Health Value subscale | 0.64 | 0.51 | - |
| Correlation with 12-item version [*r* (95% CI)] | - | 0.94 (0.93, 0.94) | 0.85 (0.84, 0.86) |
| Inter-subscale correlation: Health Consciousness [*r* (95% CI)] | - | 0.92 (0.92, 0.93) | 0.92 (0.92, 0.93) |
| Inter-subscale correlation: Health Motivation [*r* (95% CI)] | - | 0.90 (0.89, 0.90) | 0.90 (0.89, 0.90) |
| Inter-subscale correlation: Health Value [*r* (95% CI)] | - | 0.87 (0.87, 0.88) | - |

CI, confidence interval
